# Genome Assembly and Genomic Characterization of *Sanghuangporus mongolicus*

**DOI:** 10.3390/jof12090643

**Published:** 2026-08-31

**Authors:** Sitegele Wu, Haiying Bao, Tolgor Bau

**Affiliations:** 1 Jilin Province Cross-Regional Cooperation and Technological Innovation Center for the Development and Utilization of Sanghuang Resources, Jilin Agricultural University, Changchun 130118, China; sitegele118@126.com (S.W.); baohaiying2008@126.com (H.B.); 2 Key Laboratory of Edible Fungal Resources and Utilization (North), Ministry of Agriculture and Rural Affairs, Jilin Agricultural University, Changchun 130118, China

**Keywords:** *Sanghuangporus*, carbohydrate-active enzymes, genome assembly, genome annotation, secondary metabolism

## Abstract

*Sanghuangporus mongolicus* T. Bau is a wood-inhabiting medicinal fungus parasitic on *Hemiptelea davidii*, yet genomic resources for this species remain limited. Here, we generated and characterized a genome assembly from a verified monokaryotic isolate using PacBio long-read and Illumina short-read sequencing. The 34.79 Mb assembly comprised 13 contigs, with a contig N50 of 3.09 Mb and a GC content of 48.18%. BUSCO analysis recovered 98.3% complete orthologs, indicating high genome completeness. A total of 8471 protein-coding genes were predicted, including 491 genes annotated as carbohydrate-active enzymes (CAZymes), 447 transporter-related genes, and 123 cytochrome P450 genes. In addition, 16 secondary metabolite biosynthetic gene clusters were identified. A descriptive comparison with four published *Sanghuangporus* genomes showed that *S. mongolicus* had 491 CAZyme-related annotations (14.11 per Mb; 5.80 per 100 predicted protein-coding genes). Corresponding values for the other four species were 313–346 annotations, with 9.78–10.38 per Mb and 2.99–4.18 per 100 predicted genes. However, structural gene-prediction workflows differed among the five genomes, and no formal gene-family expansion analysis was conducted. Accordingly, these differences are reported descriptively and should not be interpreted as evidence of evolutionary gene-family expansion. These genomic data provide a resource for functional gene characterization and further biological studies of *S. mongolicus*.

## 1. Introduction

Wood-inhabiting fungi are major decomposers of lignocellulosic substrates and useful systems for investigating fungal diversity, substrate utilization, and functional genomic variation. The genus *Sanghuangporus* Sheng H. Wu, L.W. Zhou & Y.C. Dai (Hymenochaetales, Hymenochaetaceae) comprises poroid species distinguished from related taxa by morphological and phylogenetic evidence [1]. Research on the genus has addressed taxonomy and species diversity [1], as well as bioactive constituents [2]. However, genomic resources remain limited for several recently described species.

*Sanghuangporus mongolicus* T. Bau was originally described from basidiocarps collected on *Hemiptelea davidii* in the Horqin Sandy Land of northern China [3]. A multilocus survey of Korean specimens and culture collections subsequently reported *S. mongolicus* as a new record for Korea [4]. The species’ natural geographic and host ranges nevertheless require clarification through voucher-supported field collections. The documented Chinese collections of *S. mongolicus* occurred on *H. davidii* [3]. *H. davidii* is a characteristic tree species of the Horqin Sandy Land and an important vegetation component in this arid and semi-arid region [5]. Preliminary studies have examined the biological characteristics, cultivation, and bioactivity of *S. mongolicus* [6,7]. However, its genome structure and functional gene repertoire remain insufficiently characterized.

High-quality genome resources support the characterization of the nutritional and metabolic potential of wood-inhabiting medicinal fungi [8,9]. Relevant functional categories include carbohydrate-active enzymes (CAZymes), cytochrome P450 enzymes, transporters, and secondary metabolite biosynthetic gene clusters [8,10]. More broadly, fungal genomes provide evidence for investigating evolutionary history and diversification across the kingdom [11]. These categories inform genomic assessments of carbohydrate metabolism, nutrient transport, oxidative reactions, and specialized metabolism. Among them, CAZymes mediate the degradation, modification, and biosynthesis of carbohydrates, including plant cell-wall polysaccharides and fungal cell-wall components [12,13,14].

Genome assemblies and functional annotations have been reported for *S. sanghuang*, *S. vaninii*, *S. baumii*, and *S. weigelae* [15,16,17,18]. Jiang et al. [15] proposed, from a comparative analysis of *S. sanghuang*, *S. baumii*, and *S. vaninii*, that gene-family expansion and contraction may contribute to host specificity. Jin et al. [18] subsequently reannotated four *Sanghuangporus* genomes using a common pipeline and documented differences in CAZyme annotation counts and family composition. These studies provide a rationale for examining whether *S. mongolicus* has a distinct CAZyme-related annotation profile. We therefore hypothesized that the number and family composition of its CAZyme-related annotations would differ from those reported for other *Sanghuangporus* genomes. Because the structural gene-prediction procedure used by Jin et al. [18] differed from that used here, we evaluated this hypothesis descriptively and did not infer gene-family expansion or contraction from annotation counts alone.

In this study, we generated a genome assembly of *S. mongolicus* by integrating PacBio long-read and Illumina short-read sequencing. Genomic DNA was obtained from a verified monokaryotic isolate to reduce potential complications associated with a heterokaryotic nuclear background. We characterized the assembly quality, genome structure, and major functional gene repertoires, including CAZymes, transporters, cytochrome P450 enzymes, and secondary metabolite biosynthetic gene clusters. We also compared genome features and CAZyme-related annotations with four published *Sanghuangporus* genomes to assess the stated hypothesis at a descriptive level. The resulting genome provides a resource for further functional and biological studies of *S. mongolicus*.

## 2. Materials and Methods

### 2.1. Fungal Material and Reagents

#### 2.1.1. Strain Source and Culture Material

In September 2023, fruiting bodies of *S. mongolicus* growing on *H. davidii* were collected by Tolgor Bau in Tongliao, Inner Mongolia, China. A voucher specimen (FJAU81839) was deposited in the Fungal Herbarium of Jilin Agricultural University (FJAU). This collection was independent of the holotype designated in the original description [3]. The fruiting bodies were surface-sterilized with 95% ethanol, and small pieces of inner tissue were aseptically excised and placed on potato dextrose agar (PDA). Repeated subculturing of mycelial sectors yielded a pure isolate, designated MCCJLAU0531. The isolate was deposited in the culture collection of the Fungal Diversity Laboratory, Jilin Agricultural University (MCCJLAU). The ITS sequence of MCCJLAU0531 was analyzed using BLASTn (version 2.16.0+, National Center for Biotechnology Information, Bethesda, MD, USA) against the NCBI nucleotide (nt) database [19]. The two highest-scoring matches were *Sanghuangporus* sp. WH-2022a voucher HMJAUB7037 (GenBank accession OQ091942.1) and the holotype-derived ITS sequence of *S. mongolicus* HMJAU 67036 (NR_198365.1). Both matches showed 100% query coverage, 99.86% sequence identity, and an E-value of 0.0. The close agreement with the type-derived sequence supported the identification of MCCJLAU0531 as *S. mongolicus* (Supplementary Note S1). For routine activation, MCCJLAU0531 was cultured on PDA at 30 °C in the dark for 10 days.

#### 2.1.2. Main Reagents and Instruments

Mannitol, anhydrous magnesium sulfate, sucrose, glucose, maltose, peptone, yeast extract, and agar used for culture-medium preparation were of analytical grade. Lywallzyme was purchased from Macklin Biochemical Co., Ltd. (Shanghai, China), and 4′,6-diamidino-2-phenylindole (DAPI) was purchased from Sigma-Aldrich (St. Louis, MO, USA). The main instruments used in this study included a laminar-flow cabinet (ESCO Micro Pte Ltd, Singapore, Singapore), a constant-temperature incubator (Shanghai Yiheng Scientific Instrument Co., Ltd., Shanghai, China), an Axio Imager A2 fluorescence microscope (Carl Zeiss, Oberkochen, Germany), and a light microscope (Olympus Corporation, Tokyo, Japan).

### 2.2. Protoplast Preparation and Regeneration

#### 2.2.1. Preparation of Mycelial Pellets

Activated mycelia of *S. mongolicus* were inoculated into potato dextrose broth (PDB) containing potato infusion prepared from 200.0 g/L potato and 20.0 g/L glucose. The final volume was adjusted to 1000 mL with distilled water, and the initial pH was not adjusted. Cultures were incubated at 30 °C in the dark on a rotary shaker at 180 rpm for 6 days. The mycelia were then collected by filtration through two layers of sterile gauze and washed three times with sterile water to remove residual medium. The resulting mycelial pellets were used for protoplast preparation.

#### 2.2.2. Preparation of Protoplasts

A 0.6 mol/L mannitol solution was prepared in sterile distilled water and used as the osmotic stabilizer throughout protoplast preparation. The enzymatic solution was prepared by dissolving 0.2 g of lywallzyme in 10 mL of sterile 0.6 mol/L mannitol solution, followed by thorough mixing and filter sterilization. Fresh mycelial pellets (1.0 g fresh weight) were transferred to 10 mL of the enzymatic solution and incubated at 30 °C for 3 h, with gentle shaking every 30 min. After enzymatic digestion, a 10 μL aliquot of the digest was examined microscopically to assess protoplast release and the presence of residual hyphal material. The digest was then filtered through a sterile G2 sintered-glass filter to reduce residual hyphal fragments. The filtrate was centrifuged at 5000× *g* for 10 min, and the resulting pellet was washed three times with sterile 0.6 mol/L mannitol solution to remove residual enzyme. The final pellet was resuspended in 1–3 mL of sterile 0.6 mol/L mannitol solution to obtain the protoplast suspension.

#### 2.2.3. Preparation of Protoplast Regeneration Media

Four regeneration media were prepared with reference to previous work [20]: sucrose–peptone–yeast extract (SPY) medium, PDA supplemented with MgSO_4_, maltose–yeast extract–glucose (MYG) medium, and PDA supplemented with mannitol. SPY medium contained 109.3 g/L mannitol, 15.0 g/L sucrose, 2.0 g/L peptone, 4.0 g/L yeast extract, and 15.0 g/L agar, with the pH adjusted to 6.5. PDA supplemented with MgSO_4_ contained 72.2 g/L anhydrous MgSO_4_, potato infusion prepared from 200.0 g/L potato, 20.0 g/L glucose, and 15.0 g/L agar, with the pH adjusted to 6.5. MYG medium contained 109.3 g/L mannitol, 10.0 g/L maltose, 4.0 g/L yeast extract, 4.0 g/L glucose, and 15.0 g/L agar, with the pH adjusted to 6.5. PDA supplemented with mannitol contained 109.3 g/L mannitol, potato infusion prepared from 200.0 g/L potato, 20.0 g/L glucose, and 15.0 g/L agar, with the pH adjusted to 6.5.

#### 2.2.4. Determination of Protoplast Regeneration

Protoplasts were counted using a hemocytometer, and the suspension was adjusted to 1.0 × 10^5^ protoplasts mL^−1^ with sterile 0.6 mol/L mannitol solution. An aliquot of 100 μL was spread onto each regeneration plate, corresponding to 1.0 × 10^4^ protoplasts per plate. The plates were incubated at 30 °C in the dark, and colony counts were finalized on day 12. Apparent regeneration efficiency was calculated as the number of regenerated colonies divided by the number of plated protoplasts × 100. Because protoplast viability was not independently determined, the resulting value was considered an apparent regeneration efficiency.

Ten independent protoplast preparations (*n* = 10) were used as experimental replicates, and each preparation was evaluated on all four regeneration media. Microscopic examination and G2 filtration were used to reduce residual hyphal material before plating. However, no separate no-enzyme, osmotic-lysis, or hyphal-fragment control was included; therefore, colony formation from residual walled cells or hyphal fragments could not be quantitatively excluded.

### 2.3. Selection and Verification of the Monokaryotic Isolate

Discrete regenerated colonies ≥2 mm in diameter and showing no visible signs of colony fusion were selected and transferred individually onto PDA for subculture. After colony expansion, sterile coverslips were inserted at an angle of approximately 45° near the actively growing colony margin to allow hyphae to grow onto the coverslip surface. Hyphae were first examined by light microscopy for the presence or absence of clamp connections.

For nuclear staining, hyphae were treated with 4′,6-diamidino-2-phenylindole (DAPI; Sigma-Aldrich) at a final concentration of 1 μg/mL and incubated in the dark for 15 min. Nuclear number and distribution within septate hyphal compartments were subsequently examined by fluorescence microscopy at excitation and emission wavelengths of 358 and 461 nm, respectively. Isolates lacking clamp connections, a criterion used in protoplast-based monokaryotization of heterothallic mushrooms [21], and showing a single nucleus in each examined hyphal compartment were selected for successive subculture on PDA and re-examined using the same criteria. A verified monokaryotic isolate was subsequently selected for genomic DNA extraction and genome sequencing.

### 2.4. Genomic DNA Extraction, Library Construction, and Sequencing

The verified monokaryotic isolate was cultured on PDA at 30 °C for 6 days. Actively growing mycelia were harvested, immediately frozen in liquid nitrogen, and stored at −80 °C until DNA extraction. Genomic DNA was extracted by Novogene Bioinformatics Technology Co., Ltd. (Beijing, China) using the GP1 extraction procedure described by Wang et al. [22]. DNA purity, concentration, and integrity were assessed using a NanoDrop spectrophotometer, Qubit fluorometer, and 1% agarose gel electrophoresis, respectively.

For Illumina sequencing, a paired-end short-insert library (insert size ~350 bp) was constructed using standard procedures (DNA fragmentation, end repair, A-tailing, adapter ligation, purification, and PCR amplification). For PacBio sequencing, a SMRTbell library was prepared through DNA shearing, damage repair, end repair, adapter ligation, magnetic bead purification, and BluePippin size selection. Library quality was assessed by Qubit quantification (Thermo Fisher Scientific, Waltham, MA, USA) and Agilent 2100 Bioanalyzer analysis (Agilent Technologies, Santa Clara, CA, USA), with qPCR when required. Sequencing was performed on the Illumina PE150 (Illumina, Inc., San Diego, CA, USA) and PacBio SMRT platforms (Pacific Biosciences, Menlo Park, CA, USA) by Novogene (Beijing, China).

### 2.5. Genome Assembly and Quality Assessment

PacBio subreads were processed using CCS software (version 6.0.0, Pacific Biosciences, Menlo Park, CA, USA) [23] to generate high-accuracy circular consensus sequencing reads with the parameters min-passes = 3 and min-rq = 0.99, ensuring a predicted read accuracy of Q20 or higher. The resulting HiFi reads were used for de novo genome assembly using Hifiasm [24]. Illumina paired-end reads were used for assembly polishing and for evaluating assembly consistency based on sequencing-depth distribution. Contigs shorter than 500 bp were excluded during assembly filtering. GC content and sequencing-depth distributions were examined to identify potential anomalous contig patterns; no fixed GC-content or sequencing-depth thresholds were used for taxonomic contamination classification. Taxonomic contamination of the final assembly was further evaluated using NCBI FCS-GX (v0.5.5) [25]. The FCS-GX taxonomy report was used to assess the taxonomic assignment of individual contigs, whereas the corresponding action report was examined to identify contigs or sequence regions recommended for trimming or exclusion. Genome completeness was assessed using Benchmarking Universal Single-Copy Orthologs (BUSCO) (v6.1.0) [26,27] with the agaricomycetes_odb10 lineage dataset. BUSCO analysis was performed in genome mode using the final genome assembly of *S. mongolicus* as the input. The proportions of complete, single-copy, duplicated, fragmented, and missing BUSCOs were recorded to evaluate genome completeness.

### 2.6. Gene Prediction and Repeat Sequence Annotation

Protein-coding genes were predicted ab initio using AUGUSTUS (v2.7) [28]. No RNA-seq, full-length transcript, or homologous-protein evidence was incorporated into the gene-prediction workflow. Dispersed repetitive sequences were identified using RepeatMasker [29] and summarized according to repeat class. Tandem repeats were identified using Tandem Repeats Finder (TRF) [30]. RepeatMasker counts are reported as repeat elements, whereas TRF counts are reported as tandem-repeat loci. Minisatellite and microsatellite loci were treated as subcategories of the total tandem-repeat set. Because dispersed repeats and tandem repeats were identified separately, their genomic proportions are reported independently. tRNAs were predicted using tRNAscan-SE [31], whereas rRNAs were predicted using RNAmmer [32]. Other non-coding RNAs were identified based on the Rfam database [33] in combination with Infernal [34].

### 2.7. Functional Annotation

For functional annotation, GO terms were mapped based on the annotations of retained homologous hits obtained from databases such as NR and KOG [35]. Predicted protein sequences were searched against protein resources corresponding to KEGG [36], KOG [37], NR [38], TCDB [39], and CAZy [12] using DIAMOND [40]. Hits with an E-value ≤ 1 × 10^−5^, sequence identity ≥ 40%, and alignment coverage ≥ 40% were retained. These thresholds followed the functional annotation criteria reported by Jin et al. [18] and were adopted to maintain consistent filtering criteria for the intrageneric comparison. When multiple database matches for a predicted protein satisfied all three criteria, only the best-scoring hit was retained for annotation.

For CAZyme annotation, predicted protein sequences were searched directly against the CAZy database [12] using the same DIAMOND thresholds and best-hit selection rule. dbCAN2, dbCAN3 [41,42], or other multi-tool CAZyme-specific annotation pipelines were not applied. Secondary metabolite biosynthetic gene clusters were predicted using antiSMASH [43]. Cytochrome P450 (CYP)-related genes were annotated against the Fungal Cytochrome P450 Database (FCPD) [44].

In addition, we manually surveyed the predicted *S. mongolicus* protein set for flavonoid- and phenylpropanoid-related candidates. Targets included PAL, 4CL, CHS, CHI, F3H, FLS, and DFR based on previous reports in *Sanghuangporus* [45] and the canonical plant-type flavonoid pathway [46]. Each candidate was examined against the ClusteredNR database [38] using the NCBI Protein BLAST (BLASTp) web interface [19]. Candidate assignments were eonal annotations of highly ranked hits, query coverage, sequence identity, E-value, and alignment score. Pirin-domain-containing proteins [47] and GH78-related glycoside hydrolases [48] were also examined because characterized representatives of these groups can transform flavonoid-related compounds. This targeted survey was not intended to reconstruct a complete flavonoid biosynthetic pathway.

### 2.8. Comparative Genomic Analysis Across Sanghuangporus Species

To characterize the genomic features of *S. mongolicus*, we compared it with four *Sanghuangporus* species with published genomes: *S. sanghuang*, *S. vaninii*, *S. baumii*, and *S. weigelae*. General genome metrics included genome size, contig number, contig N50, GC content, protein-coding gene number, gene density, and reported mean gene length. We also compared available repeat-sequence proportions and the reported numbers of CAZyme-related annotations, cytochrome P450 (CYP) genes, and secondary metabolite biosynthetic gene clusters. Repeat-sequence proportions were obtained from the original studies and evaluated descriptively because annotation pipelines, databases, and classification criteria differed among studies.

For *S. mongolicus*, the predicted protein set was searched against CAZy using the DIAMOND thresholds and best-hit selection rule described above. Published CAZyme data for the other four species were obtained from Jin et al. [18]. In that study, the first three genomes were reannotated using the pipeline applied to *S. weigelae*. Because the original family-level count matrix was not publicly available, each value was manually transcribed from Figure 3 of Jin et al. [18]. The transcribed values were independently verified and cross-checked against the CAZyme class totals reported in Table 3. Values that remained ambiguous or showed unresolved inconsistencies were of Jin et al. [18] recorded as missing. The resulting matrix was treated as secondary, figure-derived data rather than as primary data from de novo CAZyme annotation.

To account for differences in genome size and predicted gene number, total CAZyme annotation counts were expressed per megabase and per 100 predicted protein-coding genes. Each species was represented by a single genome, and the counts were deterministic annotation outputs rather than replicated biological observations. Cross-species differences were therefore evaluated descriptively, without inferential significance testing. The structural gene-prediction workflow for *S. mongolicus* also differed from that used by Jin et al. [18]. Consequently, the observed differences were not interpreted as formal evidence of gene-family expansion.

### 2.9. Statistical Analysis

Ten independently conducted protoplast preparations (*n* = 10) were used as the experimental units. Each preparation was evaluated on all four regeneration media, and the medium was therefore treated as a within-preparation factor. Data are presented as the mean ± standard deviation (SD). Normality of the residuals from the repeated-measures model was assessed using the Shapiro–Wilk test [49]. Differences among the four regeneration media were analyzed using a one-way repeated-measures ANOVA. Sphericity was evaluated using Mauchly’s test [50]; when the sphericity assumption was violated (*p* < 0.05), the Greenhouse–Geisser correction [51] was applied. Significant overall effects were followed by two-sided paired t-tests with Holm adjustment [52] across the six pairwise comparisons. Partial eta-squared (ηp^2^) was calculated as the effect-size measure for the overall medium effect. Statistical analyses were performed using Python 3.12 with NumPy 2.3.5 [53] and SciPy 1.17.0 [54]. Statistical significance was defined as *p* < 0.05.

## 3. Results

### 3.1. Protoplast Regeneration and Verification of the Monokaryotic Isolate Used for Genome Sequencing

**Figure 1:**
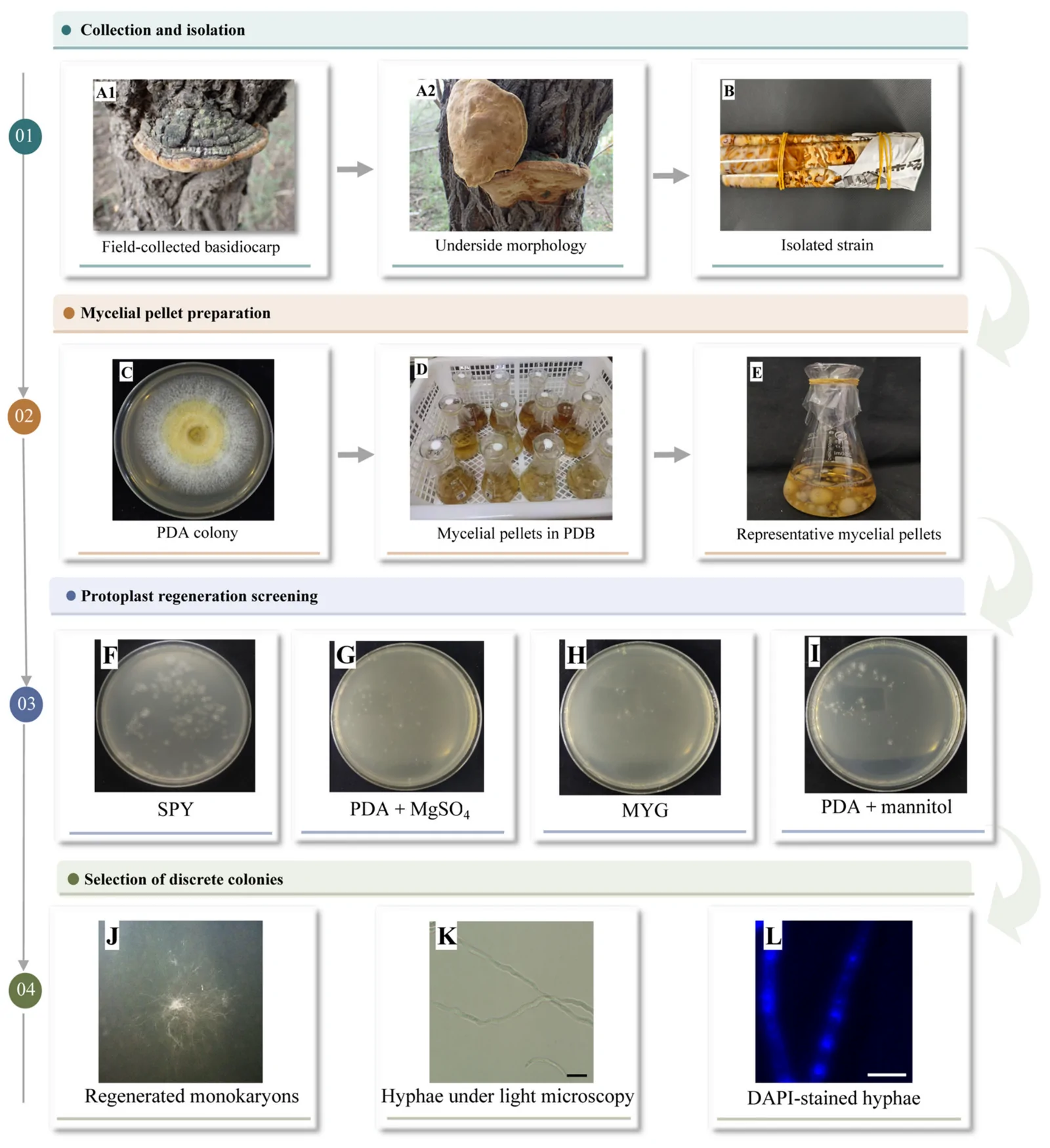
**Protoplast regeneration and verification of the monokryotic isolate used for genome sequencing in *S. mongolicus***. (**A1**,**A2**) Field-collected basidiocarp and underside morphology; (**B**,**C**) isolated culture; (**D**,**E**) mycelial pellets cultured in potato dextrose broth (PDB); (**F**–**I**) protoplast regeneration on SPY, PDA supplemented with MgSO_4_, MYG, and PDA supplemented with mannitol media, respectively; (**J**) regenerated colony selected for monokaryon verification; (**K**) hyphae observed by light microscopy, showing the absence of clamp connections; and (**L**) DAPI-stained hyphae showing a single nucleus within each examined hyphal compartment. Scale bars in (**K**,**L**) = 10 μm.; *n* = 10 independent protoplast preparations for colony-recovery analysis.

Protoplasts regenerated on all four media tested, but the numbers of regenerated colonies differed among the media. SPY medium yielded the highest colony number, with 72.1 ± 15.6 colonies per plate, followed by PDA supplemented with MgSO_4_ (54.4 ± 11.7), MYG medium (38.0 ± 8.5), and PDA supplemented with mannitol (32.2 ± 8.7). Because 1.0 × 10^4^ protoplasts were plated per dish, these values corresponded to apparent regeneration efficiencies of 0.721 ± 0.156%, 0.544 ± 0.117%, 0.380 ± 0.085%, and 0.322 ± 0.087%, respectively. The model residuals showed no evidence of departure from normality (Shapiro–Wilk test, W = 0.979, *p* = 0.642). Mauchly’s test did not indicate a violation of sphericity (W = 0.809, χ^2^(5) = 1.636, *p* = 0.897); therefore, no correction to the degrees of freedom was required. Regeneration medium significantly affected colony recovery (one-way repeated-measures ANOVA, F(3, 27) = 39.18, *p* = 5.646 × 10^−10^, partial η^2^ = 0.813), indicating a large medium effect. Holm-adjusted paired comparisons showed that colony recovery on SPY medium was higher than that on PDA supplemented with MgSO_4_ (adjusted *p* = 0.0112), MYG medium (adjusted *p* = 1.10 × 10^−4^), and PDA supplemented with mannitol (adjusted *p* = 3.52 × 10^−5^). PDA supplemented with MgSO_4_ also yielded higher colony recovery than MYG medium (adjusted *p* = 0.0112) and PDA supplemented with mannitol (adjusted *p* = 4.05 × 10^−4^), whereas MYG medium and PDA supplemented with mannitol did not differ significantly (adjusted *p* = 0.118). Thus, under the conditions tested, SPY medium supported the highest protoplast regeneration among the four media evaluated (Figure 1A–I).

Discrete regenerated colonies were selected for subculture and micro scopic examination. Numbers 1–4 indicate the four main experimental stages: (01) collection and isolation, (02) mycelial pellet preparation, (03) protoplast regeneration screening, and (04) selection of discrete colonies. No clamp connections were observed by light microscopy, and DAPI staining revealed a single nucleus in each examined hyphal compartment. These morphological and nuclear characteristics were retained after successive subcultures on PDA. On this basis, a verified monokaryotic isolate that met the morphological and nuclear criteria was selected for genomic DNA extraction and subsequent genome sequencing (Figure 1J–L).

### 3.2. Genome Assembly and Structural Features of S. mongolicus

A genome assembly of *S. mongolicus* was generated by integrating PacBio long-read and Illumina short-read sequencing. Agarose gel electrophoresis showed that the predominant genomic DNA signal migrated above the 20 kb marker, consistent with the presence of high-molecular-weight DNA. NanoDrop and Qubit measurements yielded DNA concentrations of 175.659 ng/μL and 122 ng/μL, respectively, indicating that the DNA quality was sufficient for subsequent library construction and sequencing (Figure S1). The whole-genome sequencing project has been deposited in NCBI under BioProject accession number PRJNA1481442 and BioSample accession number SAMN61081292. The genome assembly has been released in GenBank under accession number JCAKBH000000000.

**Figure 2:**
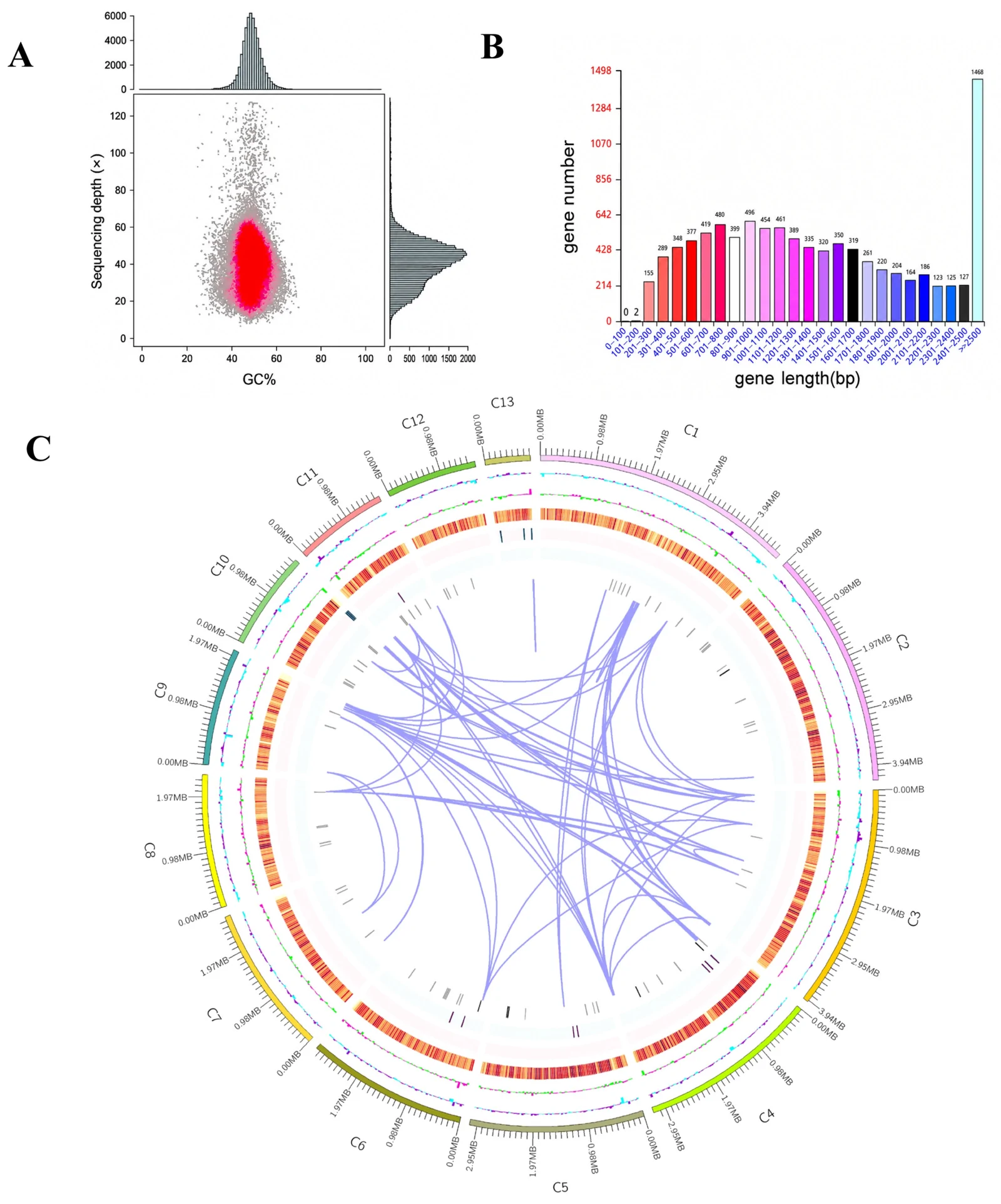
**Genome assembly and structural features of *S. mongolicus*.** (**A**) Distribution of GC content and sequencing depth across the assembled genome. (**B**) Distribution of predicted gene lengths. (**C**) Circular representation of the *S. mongolicus* genome. The outermost circle indicates the positional coordinates of the 13 contigs. From outside to inside, the tracks represent GC content, GC skew, gene density, and intragenomic syntenic links. GC skew was calculated as (G − C)/(G + C).

The final assembly of the *S. mongolicus* genome was 34,792,067 bp in total length, with a GC content of 48.18%. The assembly consisted of 13 contigs, with the longest contig reaching8 4,638,348 bp and a contig N50 of 3,090,157 bp. Individual contig lengths ranged from 802,757 to 4,638,348 bp, with the complete per-contig assembly statistics provided in Table S1. AUGUSTUS predicted 8471 protein-coding genes in the *S. mongolicus* genome. The total predicted gene length was 13,644,592 bp, corresponding to 39.22% of the genome, and the mean predicted gene length was 1611 bp (Table 1). Examination of GC content and sequencing-depth distributions showed no distinct contig cluster with an anomalous GC–depth pattern (Figure 2).

Taxonomic contamination was further assessed using NCBI FCS-GX, which from outer to inner, the six layers represent: >1 Mb contigs; G+C content in 1-kb windows (blue, below average; purple, above average); GC skew (green, <1; pink, >1); gene density (coding genes/rRNA/snRNA/tRNA; darker orange indicates higher density); innermost repeats (purple arcs connecting homologous segments >8 kb with ≥90% identity). inferred Basidiomycetes as the primary taxonomic division for the assembly. All 13 contigs were assigned to this primary division, with per-contig coverage by the basidiomycete division ranging from 88% to 96%. The aggregate taxonomic coverage was 93.72%. The corresponding FCS-GX action report contained no flagged records, and no contigs or sequence regions were recommended for trimming or exclusion. Thus, FCS-GX detected no non-target contamination requiring removal from the final genome assembly. BUSCO analysis recovered 98.3% complete orthologs, including 98.0% single-copy and 0.3% duplicated BUSCOs. Fragmented and missing BUSCOs accounted for 0.1% and 1.6%, respectively, indicating that most of the expected conserved Agaricomycetes gene space was represented in the assembly.

RepeatMasker identified 3355 dispersed repetitive elements spanning 1,157,262 bp, accounting for 3.3262% of the genome. These included 2444 LTR retrotransposon elements, 284 DNA transposon elements, 584 LINEs, 15 SINEs, 18 rolling-circle elements, and 10 unclassified repeat elements. LTR retrotransposons represented the predominant dispersed-repeat category, spanning 1,030,193 bp and accounting for 2.9610% of the genome.

TRF identified 3471 tandem-repeat loci spanning 287,732 bp, accounting for 0.8270% of the genome. Among these, 2330 loci were classified as minisatellites, spanning 136,764 bp (0.3931% of the genome), and 543 loci were classified as microsatellites, spanning 21,480 bp (0.0617%). The remaining 598 loci, spanning 129,488 bp (0.3722% of the genome), belonged to the total tandem-repeat set but were not included in the minisatellite or microsatellite subclasses.

### 3.3. Overview of Functional Annotation

#### 3.3.1. Go, Kegg, Kog, and Nr Annotation

**Figure 3:**
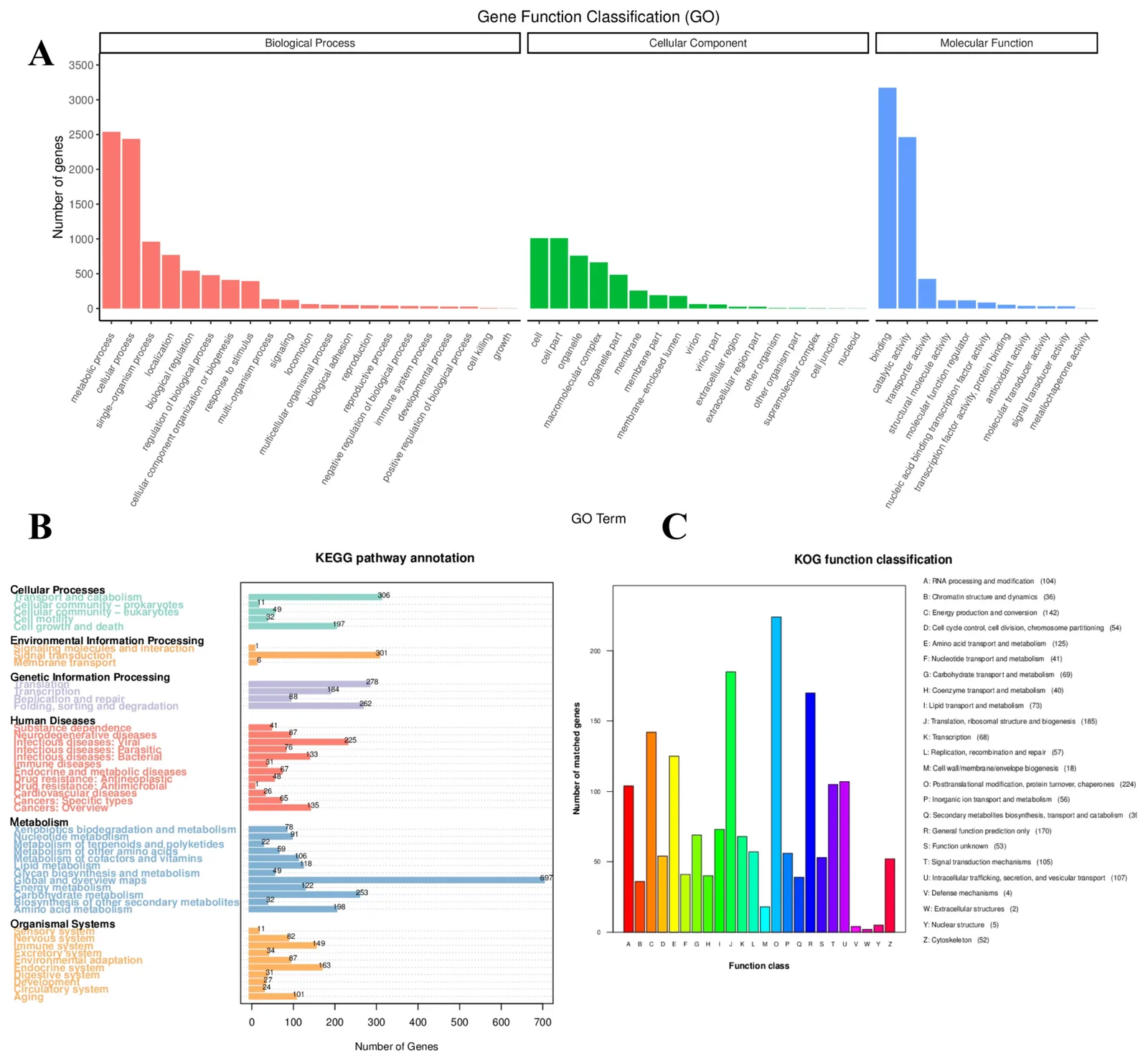
**Functional annotation overview of the *S. mongolicus* genome:** (**A**) GO annotation; (**B**) KEGG annotation; (**C**) KOG annotation.

GO annotation assigned functional categories to 5417 genes, spanning 49 subcategories across the biological process, molecular function, and cellular component domains. Within biological process, metabolic process, cellular process, and single-organism process were among the most highly represented terms. Binding-related terms predominated in molecular function, whereas cell- and organelle-related terms predominated in cellular component. Of the 6766 genes with KEGG annotations, 1825 were assigned to metabolism-related pathways, accounting for 27.0%. Most of these genes were associated with global and overview maps, carbohydrate metabolism, or amino acid metabolism. KOG annotation further classified 1664 genes into 26 functional categories. The most abundant category was posttranslational modification, protein turnover, and chaperones, followed by translation, ribosomal structure, and biogenesis (Figure 3). NR homology searches identified *S. baumii* as the best-hit species for 6362 genes, the highest total among all matched species (Figure S2).

#### 3.3.2. Cazyme Family Composition

**Figure 4:**
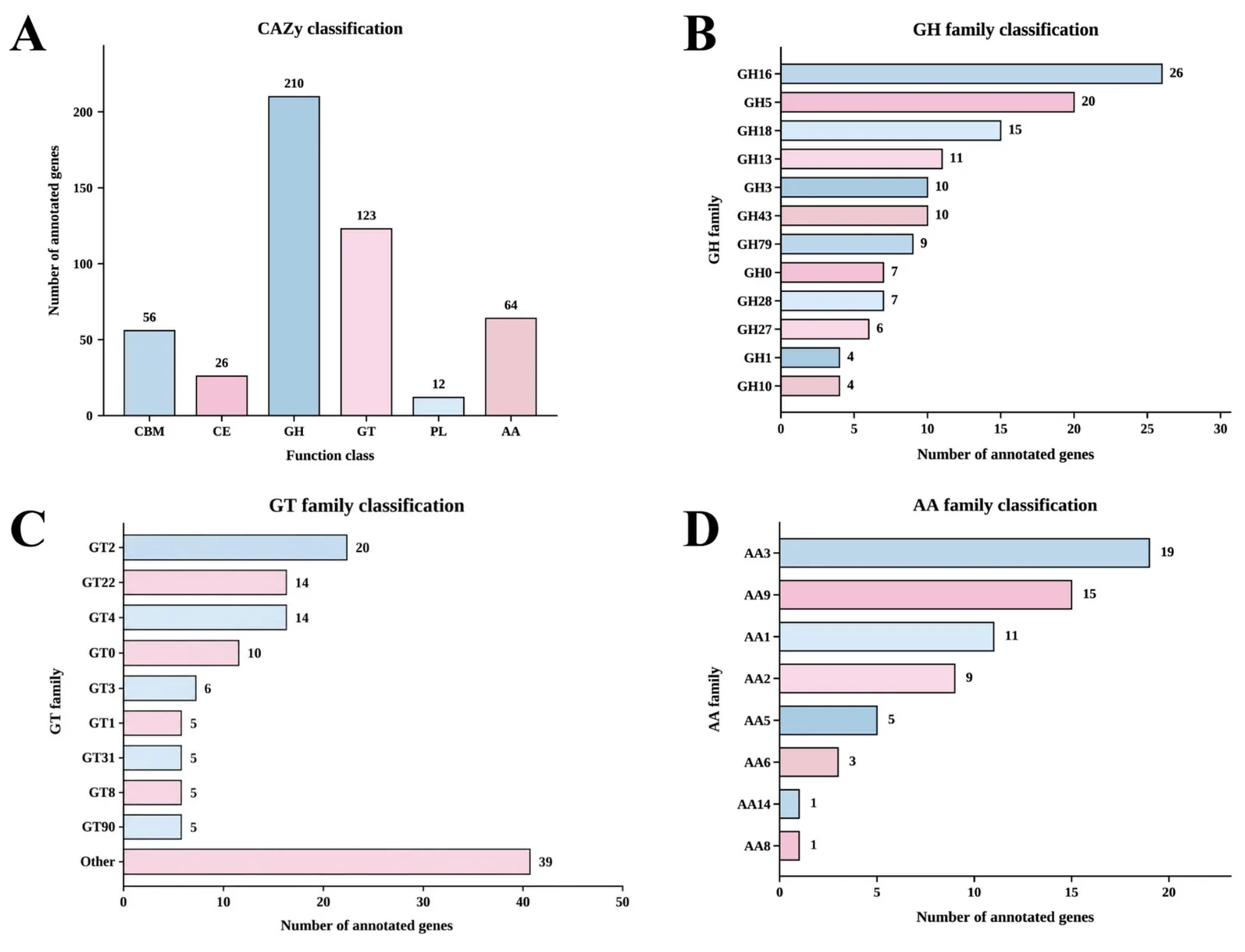
**Distribution of carbohydrate-active enzyme (CAZyme)-related annotations in *S. mongolicus*:** (**A**) Distribution across the major CAZyme classes; (**B**–**D**) Family-level distributions of glycoside hydrolases (GHs), glycosyltransferases (GTs), and auxiliary activities (AAs) respectively.

Using the DIAMOND–CAZy criteria described above, 491 predicted protein-coding genes were annotated as CAZyme-related genes, including 210 glycoside hydrolases (GHs), 123 glycosyltransferases (GTs), 64 auxiliary activity enzymes (AAs), 56 carbohydrate-binding modules (CBMs), 26 carbohydrate esterases (CEs), and 12 polysaccharide lyases (PLs). Among these categories, GHs represented the largest group, followed by GTs and AAs (Figure 4).

Within the GH class, GH16, GH5, GH18, GH13, GH3, and GH43 were among the most frequently annotated families. Members of GH5 [55], GH3 [13], and GH43 [56] include enzymes associated with the processing of cellulose-, hemicellulose-, and oligosaccharide-derived substrates. GH16 β-(1,3)-glucanases participate in fungal cell-wall morphogenesis [57], whereas GH18 chitinases contribute to cell-wall remodeling and, in some fungi, defense and mycoparasitic interactions [58]. Therefore, the observed family composition indicates a diverse set of predicted carbohydrate-related functions but does not establish the specific physiological or ecological roles of these genes. The higher CAZyme annotation counts in *S. mongolicus* were distributed across several functional classes rather than being restricted to a single class.

#### 3.3.3. Transporter Annotation

**Figure 5:**
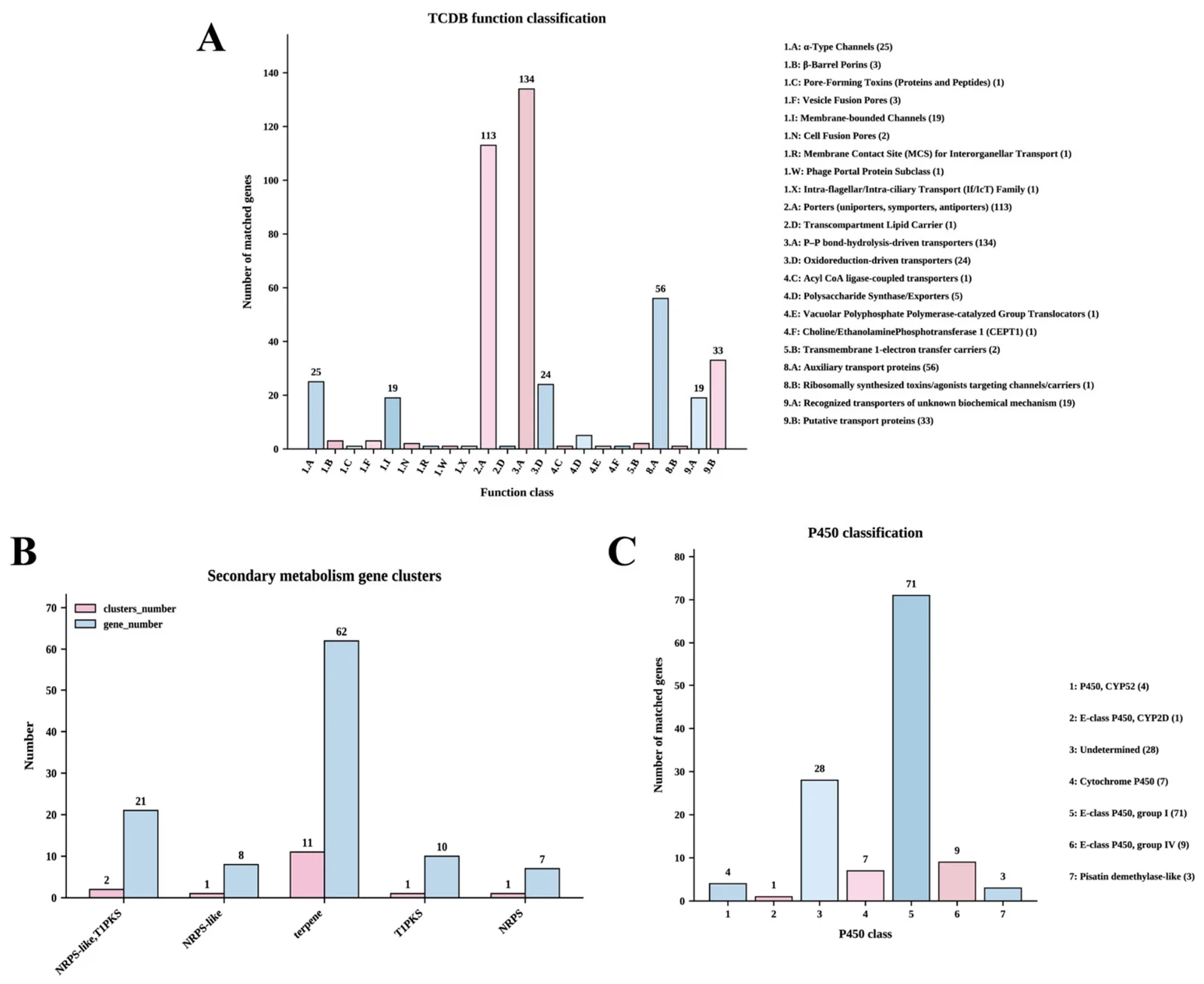
**Functional annotation profiles of *S. mongolicus*:** (**A**) TCDB transporter classification; (**B**) secondary metabolite biosynthetic gene clusters; (**C**) CYP450 classification.

Annotation against the TCDB database [39] identified 447 transporter-related genes. Among these, P–P-bond-hydrolysis-driven transporters represented the largest category, comprising 134 genes, followed by porters, with 113 genes. These results suggest that *S. mongolicus* possesses a diverse repertoire of transport systems potentially involved in ion transport, nutrient uptake, and metabolite efflux (Figure 5A).

### 3.4. Analysis of Cyp450 Genes and Secondary Metabolite Biosynthetic Gene Clusters

AntiSMASH analysis predicted 16 secondary metabolite biosynthetic gene clusters involving 108 functional genes and covering 5 major cluster types. Terpene gene clusters were the most abundant, with 11 clusters identified. Further integration with KEGG pathway annotation revealed 13 genes encoding key enzymes associated with the terpenoid backbone biosynthesis pathway (map00900), including acetyl-CoA C-acetyltransferase, hydroxymethylglutaryl-CoA synthase, hydroxymethylglutaryl-CoA reductase, diphosphomevalonate decarboxylase, isopentenyl-diphosphate isomerase, and farnesyl diphosphate synthase (Table 2). These results suggest that *S. mongolicus* contains predicted genes consistent with potential for isoprenoid precursor synthesis and terpene backbone formation. In addition to terpene clusters, NRPS-like, T1PKS, and hybrid gene clusters were also identified (Figure 5B).

CYP450 annotation identified 123 related genes in the *S. mongolicus* genome. Among them, E-class P450 group I represented the predominant category, comprising 71 genes and accounting for 57.7% of the total. In addition, 28 genes could not be assigned to known CYP categories, representing 22.76% of all annotated CYP450 genes (Figure 5C).

Targeted screening of the predicted protein set identified a phenylalanine ammonia-lyase (PAL) candidate encoded by A1670 and a 4-coumaroyl-CoA ligase-like (4CL-like) candidate encoded by A5498. Manual NCBI BLASTp examination against the ClusteredNR database provided strong sequence-similarity support for these assignments: A1670 showed 100% query coverage and 96.16% identity to an annotated PAL from *S. baumii*, whereas A5498 showed 100% query coverage and 93.06% identity to an annotated 4-coumaroyl-CoA ligase from *S. baumii* (Table S2).

A pirin-domain-containing protein candidate (A4080) and two GH78-related glycoside hydrolase candidates (A2183 and A6830) were also identified and supported by BLASTp similarity to annotated fungal homologs (Table S2). In contrast, the targeted screening did not recover candidates corresponding to several downstream enzymes of the canonical plant-type flavonoid biosynthetic pathway, including CHS, CHI, F3H, FLS, and DFR. None of the identified candidate genes was located within the predicted secondary metabolite biosynthetic gene clusters. Thus, the genomic survey identified proteins related to upstream phenylpropanoid metabolism and proteins potentially associated with flavonoid-compound transformation, but it did not provide evidence of a complete canonical plant-type flavonoid biosynthetic pathway in *S. mongolicus*.

### 3.5. Intrageneric Comparative Genomic Analysis of Sanghuangporus

To clarify the genomic features of *S. mongolicus* within the genus *Sanghuangporus*, we compared five *Sanghuangporus* genomes, including *S. mongolicus*, *S. baumii*, *S. sanghuang*, *S. vaninii*, and *S. weigelae*. The genome structure and functional annotation profiles of five *Sanghuangporus* species are summarized in Table S3. The genome sizes of these five species were relatively similar, ranging from 31.64 to 34.79 Mb, with *S. mongolicus* having the largest genome (34.79 Mb). In terms of assembly contiguity, both *S. mongolicus* and *S. weigelae* consisted of 13 contigs, whereas *S. mongolicus* showed the highest contig N50 value, at 3.09 Mb. GC content varied only slightly among the species, ranging from 47.25% to 48.18%. The number of predicted protein-coding genes ranged from 8278 to 11,310 among the five *Sanghuangporus* genomes, corresponding to gene densities of 243.5–327.6 genes/Mb (Table S3). *S. mongolicus* contained 8471 predicted protein-coding genes and had a gene density of 243.5 genes/Mb, the lowest among the five compared genomes. For species with available data, reported mean gene lengths ranged from 1597 bp in *S. sanghuang* to 2061 bp in *S. weigelae*, whereas the mean predicted gene length of *S. mongolicus* was 1611 bp. Thus, the overall gene number, gene density, and mean gene length of *S. mongolicus* did not show obvious increases relative to the compared congeners. However, these comparisons should be interpreted descriptively because the published genomes were annotated using different pipelines.

Repeat-sequence proportions also varied among the available *Sanghuangporus* genome annotations (Table S3). In S. mongolicus, dispersed repeats accounted for 3.3262% of the genome, which was close to the 2.90% reported for *S. sanghuang*. LTR retrotransposons accounted for 2.9610% of the *S. mongolicus* genome, similar to the 2.72% reported for *S. sanghuang*, but lower than the reported values for *S. vaninii* (9.84%) and *S. weigelae* (12.22%). Tandem repeats accounted for 0.8270% of the *S. mongolicus* genome, close to the 0.85% reported for *S. sanghuang* and higher than the 0.27% reported for both *S. vaninii* and *S. weigelae*. These values indicate substantial variation in annotated repeat abundance and composition among the available *Sanghuangporus* genomes. Because different studies used different repeat-annotation pipelines, databases, and classification criteria, these comparisons are descriptive.

Comparative analysis of CAZyme annotations showed that *S. mongolicus* contained 491 CAZyme-related genes, compared with 313 in *S. baumii*, 346 in *S. sanghuang*, 338 in *S. vaninii*, and 332 in *S. weigelae* (Table S3). After normalization by genome size, *S. mongolicus* had 14.11 CAZyme annotations/Mb, whereas the other four species ranged from 9.78 to 10.38 annotations/Mb. Normalization by predicted protein-coding gene number showed a similar pattern: *S. mongolicus* contained 5.80 CAZyme annotations per 100 predicted genes, compared with 2.99–4.18 among the other four species. Thus, the higher CAZyme annotation count in *S. mongolicus* was retained after normalization for both genome size and predicted gene number.

**Figure 6:**
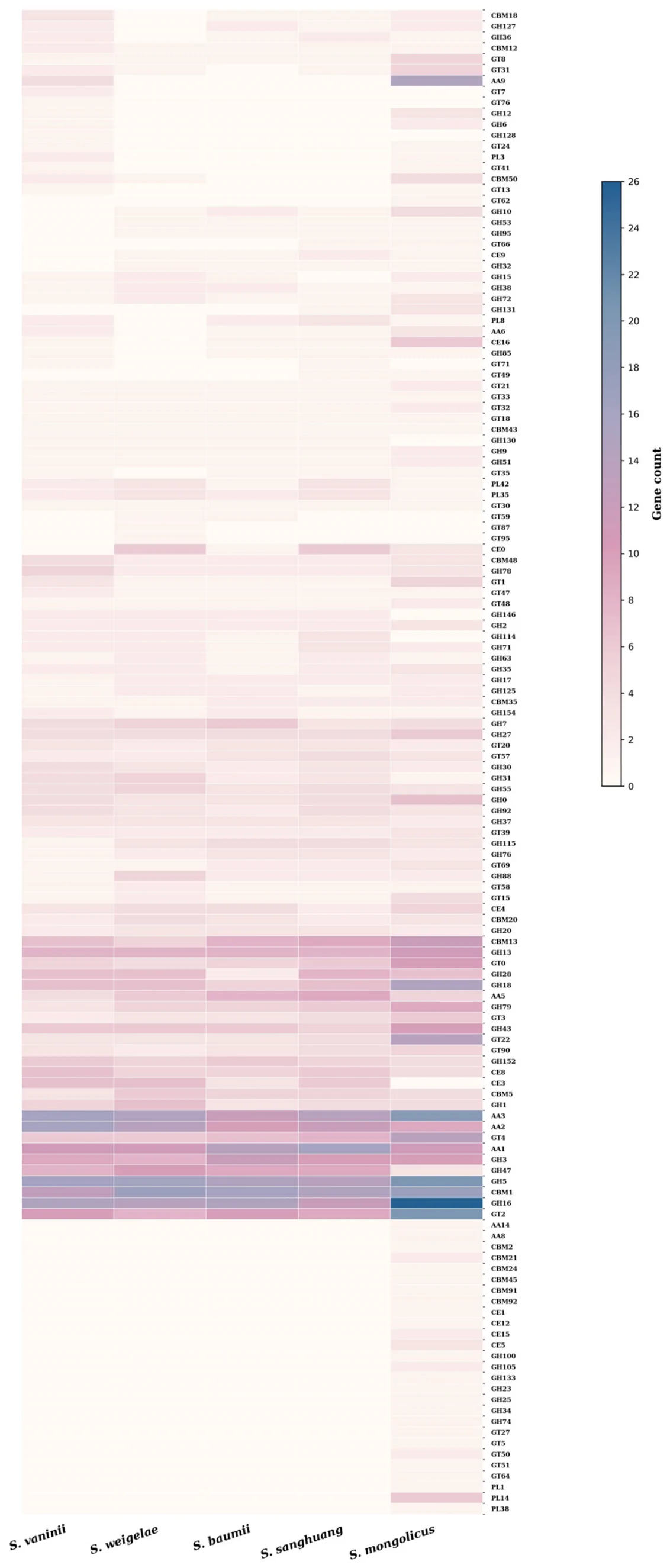
**Heatmap of CAZyme family gene counts across five *Sanghuangporus* species.** Rows represent CAZyme families, columns represent the five species, and colour intensity indicates the number of genes. Data for *S. mongolicus* were generated through the CAZyme annotation performed in this study, whereas data for the other four species were manually transcribed from Jin et al. [18].

At the CAZyme-class level, *S. mongolicus* also had the highest absolute annotated counts among the five species for CBMs, CEs, GHs, GTs, PLs, and AAs, with 56, 26, 210, 123, 12, and 64 genes, respectively (Figure 6). These higher counts were therefore distributed across several CAZyme classes rather than being attributable to a single class. Because each species was represented by a single genome and the structural gene-prediction workflows were not identical across all five genomes, these normalized and absolute differences are interpreted descriptively and were not subjected to inferential significance testing or interpreted as evidence of evolutionary gene-family expansion.

Comparative analysis of CYP450 annotation showed that the total number of CYP450-related genes among the five species ranged from 112 to 136, with *S. vaninii* having the highest number (136) and *S. mongolicus* containing 123. E-class P450 group I was the predominant category in all five species. Terpene clusters were the predominant cluster type, with counts ranging from 9 to 12 per genome, whereas total cluster counts ranged from 16 to 19. In *S. mongolicus*, 16 secondary metabolite biosynthetic gene clusters were predicted, including NRPS-like/T1PKS hybrid clusters and NRPS-like clusters. Overall, *S. mongolicus* showed high assembly contiguity and higher absolute and normalized CAZyme annotation counts than the four congeners examined.

## 4. Discussion

Establishing a monokaryotic culture provided a defined source of genomic DNA for assembly. Among the four media tested, SPY produced the highest colony recovery from an identical input of 1.0 × 10^4^ protoplasts. It yielded approximately 1.33-, 1.90-, and 2.24-fold more colonies than PDA supplemented with MgSO_4_, MYG, and PDA supplemented with mannitol, respectively. This larger recovered-colony pool could facilitate subsequent isolate selection and screening. However, the experiment did not identify the responsible medium component or establish SPY as optimal beyond the tested conditions. The selected isolate lacked clamp connections and contained a single nucleus in each examined hyphal compartment, supporting its use for genome sequencing.

The genome size, GC content, and predicted gene number of *S. mongolicus* were comparable to those reported for other *Sanghuangporus* species. BUSCO analysis recovered 98.3% complete orthologs, indicating the high completeness of the present assembly. Published values were 94.99% [18], whereas a later study reported 91.0–93.7% for newly assembled genomes of *S. baumii*, *S. sanghuang*, *S. vaninii*, and *S. weigelae* [59]. These comparisons support the high recovery of conserved genes but do not establish a strict quality ranking. The studies differed in BUSCO versions, lineage datasets, sequencing strategies, and genome-processing workflows.

Repeat analysis showed that LTR retrotransposons were the predominant dispersed repeats in *S. mongolicus*, accounting for 2.9610% of the genome. Its total dispersed-repeat proportion (3.3262%) was similar to that of *S. sanghuang* (2.90%), whereas higher LTR proportions were reported for *S. vaninii* and *S. weigelae*. This variation may reflect differences in repeat accumulation and turnover among lineages. However, comparisons remain descriptive because repeat-detection pipelines and classification criteria differed among studies.

The clearest functional pattern in the intrageneric comparison was the higher number of CAZyme-related annotations in *S. mongolicus*. The genome contained 14.11 annotations/Mb and 5.80 annotations per 100 predicted protein-coding genes. The corresponding ranges among the other four species were 9.78–10.38 annotations/Mb and 2.99–4.18 annotations per 100 genes. After normalization by genome size and predicted gene number, the higher CAZyme annotation count in *S. mongolicus* remained apparent in these descriptive metrics. Nevertheless, it represents a difference in annotated repertoire size, not evidence of evolutionary gene-family expansion. Structural gene-prediction workflows differed among genomes, and no phylogenetically informed expansion analysis was performed. The CAZyme family composition suggests a diverse predicted capacity for carbohydrate processing. GH5, GH3, and GH43 include enzymes acting on plant-derived polysaccharides and oligosaccharides [13,55,56]. GH16 and GH18 include proteins involved in fungal cell-wall metabolism [57,58]. Family membership alone, however, does not establish the substrate specificity or activity of individual proteins. Comparative genomic studies have revealed substantial variation in lignocellulose-degrading enzyme repertoires among wood-decay fungi, and the conventional white-rot/brown-rot dichotomy does not fully predict their enzymatic complements [60,61,62]. Total CAZyme number likewise does not determine decay strategy or degradation efficiency. In addition, CAZymes were identified by DIAMOND searches against CAZy rather than by consensus predictions from dbCAN2 or dbCAN3 [41,42]. Counts may therefore vary with gene-model quality, database release, similarity thresholds, and annotation strategy. The 491 annotations should consequently be interpreted as a relatively large predicted repertoire within this comparison, not as evidence of greater lignocellulose-degrading capacity. Uniform reannotation, expression profiling, secretome analysis, and biochemical assays are needed to determine the functions of these genes.

The predicted transporters, CYP450s, and secondary metabolite biosynthetic gene clusters extend this functional inventory beyond carbohydrate metabolism. Transporter-related genes may mediate nutrient uptake, ion homeostasis, and metabolite efflux [39]. The genome contained 123 CYP450-related genes, predominantly from E-class P450 group I. Fungal CYP450s commonly contribute to the oxidative metabolism of lipids, sterols, aromatic compounds, and xenobiotics [63]. Terpene clusters predominated among the predicted secondary metabolite biosynthetic gene clusters, and genes involved in terpenoid backbone biosynthesis were also detected. Together, these features indicate genomic potential for terpene precursor synthesis and oxidative modification, although they do not demonstrate pathway activity.

The targeted screening did not support a complete canonical flavonoid biosynthetic pathway in *S. mongolicus*. Previous studies have reached different conclusions regarding endogenous flavonoid production in fungi. The absence of key canonical enzymes has been used to argue against conventional biosynthesis in mushrooms [64]. Conversely, flavonoid-associated metabolites and PAL expression have been reported in *S. baumii* [45]. We identified PAL- and 4CL-like candidates with high similarity to annotated *Sanghuangporus* homologs, but did not recover CHS, CHI, F3H, FLS, or DFR candidates. PAL- and 4CL-related proteins alone therefore do not establish a complete pathway. Characterized representatives of pirin-domain proteins [47] and GH78 α-L-rhamnosidases [48] can transform flavonoid-related compounds, but family membership and sequence similarity cannot define the specific activities of the candidates identified here. A non-canonical NRPS–PKS-based pathway has been demonstrated for chlorflavonin biosynthesis in a fungus [65], but the present genome provided no specific evidence linking an analogous system to flavonoid formation. Direct metabolomic and biochemical analyses are required to establish endogenous flavonoid production in *S. mongolicus*.

Interpretation of all predicted gene repertoires is constrained by the structural annotation strategy. Protein-coding genes were predicted ab initio using AUGUSTUS without transcriptomic or homologous-protein evidence. Some models may therefore be false positives or contain inaccurate exon–intron boundaries. Similar gene counts, gene densities, and mean gene lengths among *Sanghuangporus* genomes do not validate individual models because the annotation pipelines differed. Future refinement should incorporate transcript and homologous-protein evidence using an evidence-supported workflow such as BRAKER3 [66], MAKER2 [67], or an equivalent pipeline. Despite this limitation, the assembly and functional annotations provide a genomic resource for targeted gene characterization and further biological studies of *S. mongolicus*.

## 5. Conclusions

In this study, we generated a 34.79 Mb genome assembly of *S. mongolicus*, comprising 13 contigs and 8471 predicted protein-coding genes. Functional annotation identified 491 CAZyme-related genes, 447 transporter-related genes, 123 cytochrome P450 genes, and 16 secondary metabolite biosynthetic gene clusters. In the descriptive intrageneric comparison, *S. mongolicus* showed higher absolute and normalized CAZyme annotation counts than the four congeners examined. The genome presented here provides a resource for the subsequent functional characterization of *S. mongolicus*. Transcriptomic, secretomic, and biochemical analyses will be required to determine the functions of the annotated genes.

## Figures and Tables

**Table 1:** Genome-wide features of *S. mongolicus*.

Statistics	*S. mongolicus*
Contig number	13
Max contig length (bp)	4,638,348
Contig N50 (bp)	3,090,157
Total contig length (bp)	34,792,067
GC content (%)	48.18
Number of protein-coding genes	8471
Total predicted gene length (bp)	13,644,592
Predicted gene length/genome (%)	39.22
Mean predicted gene length (bp)	1611
Total dispersed repeat elements (n)	3355
LTR retrotransposon elements (n)	2444
DNA transposon elements (n)	284
LINE elements (n)	584
SINE elements (n)	15
Rolling-circle repeat elements (n)	18
Unclassified dispersed repeat elements (n)	10
Total tandem-repeat loci (n)	3471
Minisatellite loci (n)	2330
Microsatellite loci (n)	543
Tandem-repeat loci outside the minisatellite/microsatellite subclasses (n) ^a^	598

Note: Counts of dispersed repeats represent repeat elements identified by RepeatMasker, whereas tandem-repeat counts represent loci identified by Tandem Repeats Finder. Minisatellite and microsatellite loci are subcategories of the 3471 total tandem-repeat loci. ^a^ The remaining 598 loci were calculated as 3471 − 2330 − 543.

**Table 2:** Key Enzymes Involved in Terpenoid Backbone Biosynthesis (KEGG Map00900) in *S. mongolicus*.

Gene ID	KO Term	Gene Name and Definition	EC Number
A1840	K00804	GGPS1; geranylgeranyl diphosphate synthase, type III	2.5.1.1; 2.5.1.10; 2.5.1.29
A2763	K05906	PCYOX1; prenylcysteine oxidase/farnesylcysteine lyase	1.8.3.5; 1.8.3.6
A3111	K01823	idi, IDI; isopentenyl-diphosphate Delta-isomerase	5.3.3.2
A3910	K01641	HMGCS; hydroxymethylglutaryl-CoA synthase	2.3.3.10
A4601	K11778	DHDDS, RER2, SRT1; ditrans, polycis-polyprenyl diphosphate synthase	2.5.1.87
A5710	K00626	ACAT, atoB; acetyl-CoA C-acetyltransferase	2.3.1.9
A5812	K00587	ICMT, STE14; protein-S-isoprenylcysteine O-methyltransferase	2.1.1.100
A6634	K01597	MVD, mvaD; diphosphomevalonate decarboxylase	4.1.1.33
A7795	K05955	FNTA; protein farnesyltransferase/geranylgeranyltransferase type-1 subunit alpha	2.5.1.58; 2.5.1.59
A8119	K05954	FNTB; protein farnesyltransferase subunit beta	2.5.1.58
A8168	K06013	STE24; STE24 endopeptidase	3.4.24.84
A1132	K00021	HMGCR; hydroxymethylglutaryl-CoA reductase (NADPH)	1.1.1.34
A1308	K00787	FDPS; farnesyl diphosphate synthase	2.5.1.1; 2.5.1.10

## Data Availability

The genome sequencing data generated in this study have been deposited in NCBI under BioProject accession number PRJNA1481442 and BioSample accession number SAMN61081292. The whole-genome assembly has been released in GenBank under accession number JCAKBH000000000. Other data supporting the findings of this study are available within the article and its Supplementary Materials. Further inquiries can be directed to the corresponding author.

## Supplementary Materials

The following supporting information can be downloaded at https://www.mdpi.com/article/10.3390/jof12090643/s1. Supplementary Note S1; Figure S1. Agarose gel electrophoresis of genomic DNA from *S. mongolicus*; Figure S2. NR Annotation of *S. mongolicus*; Table S1. Lengths of individual contigs in the final *S. mongolicus* genome assembly; Table S2. Manual NCBI BLASTp verification of flavonoid- and phenylpropanoid-related candidate proteins identified in *S. mongolicus*; Table S3. Comparison of genome features, repeat-sequence content, and selected functional annotations among five *Sanghuangporus* species.
