# Supplementary material for: Genome Assembly and Genomic Characterization of Sanghuangporus mongolicus
Source: J Fungi (Basel). 2026 Aug 31;12(9):643. doi: 10.3390/jof12090643 (PMC13608065; doi:10.3390/jof12090643)
Supplement: Supplementary file 1 [file jof-12-00643-s001.zip › jof-4441960-supplementary.pdf]

Supplementary Note S1:

ITS:GGAAGGATCATTATCGAGTTTTTGAAAGCGAGGCTTGCTGCTGGCGCAAGTTAATT
TTTGCGCATGTGCACGGTCTTCGCGCTCAAGTCCCAACTCAAACCCCTGTGCACCTTAT
CATATCGCGAGTCGAAGTTAGTAGTCTGAGGTGGGTCTCGTAAGTAATTGTTAGTTAGT
AGAAGGGGCGAAAGCGAGTCTTTGCTCGTTAGGTAACCCTTTCGAAAAGTCAATGAA
AGCGAGCGACGTCGGGTGAAGGCTTTCGGCTTGTCGTTACAAACCCCTTATATTGTCTT
TGTGAATGTAATGCTCCTCGTGGGCGAAAATAAATACAACTTTCAACAACGGATCTCTT
GGCTCTCGCATCGATGAAGAACGCAGCGAAATGCGATAAGTAATGTGAATTGCAGAAT
TCAGTGAATCATCGAATCTTTGAACGCACCTTGCGCCCCTTGGTATTCCGAGGGGCATG
CCTGTTTGAGTGTCATGTTTATCTCAAACCGCTCGTCTTTTCTTAATCGGAAGGGCTTG
AGGTTTGGACTTGGAGGTTTTACTGCTGGCGCCTTTCGAGGGGTCGGCTCCTCTTAAAT
ACATTAGCTGGGCTTTGGCTCGCGTTTACGGTGTAATAGTTGATTCCGTTCACCAACGA
GCGCTTGCCTGACGAGCTCGCTTCTAACCGTCCGCGTCGTCGGACAAGGAGTGTTACC
CCTCCTTCTTGACACCTTTGACCTCAAA

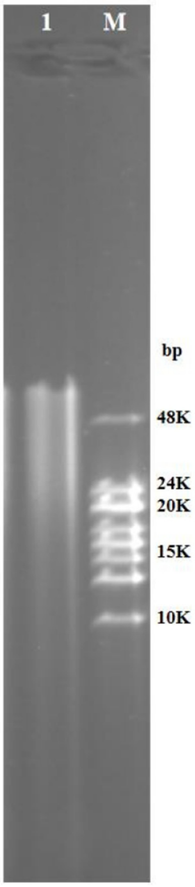

Figure S1. Agarose gel electrophoresis of genomic DNA from *S. mongolicus*. Lane 1, genomic DNA sample; lane M, high-molecular-weight DNA marker (10, 15, 20, 24, and 48 kb). The predominant genomic DNA signal migrated above the 20-kb marker.

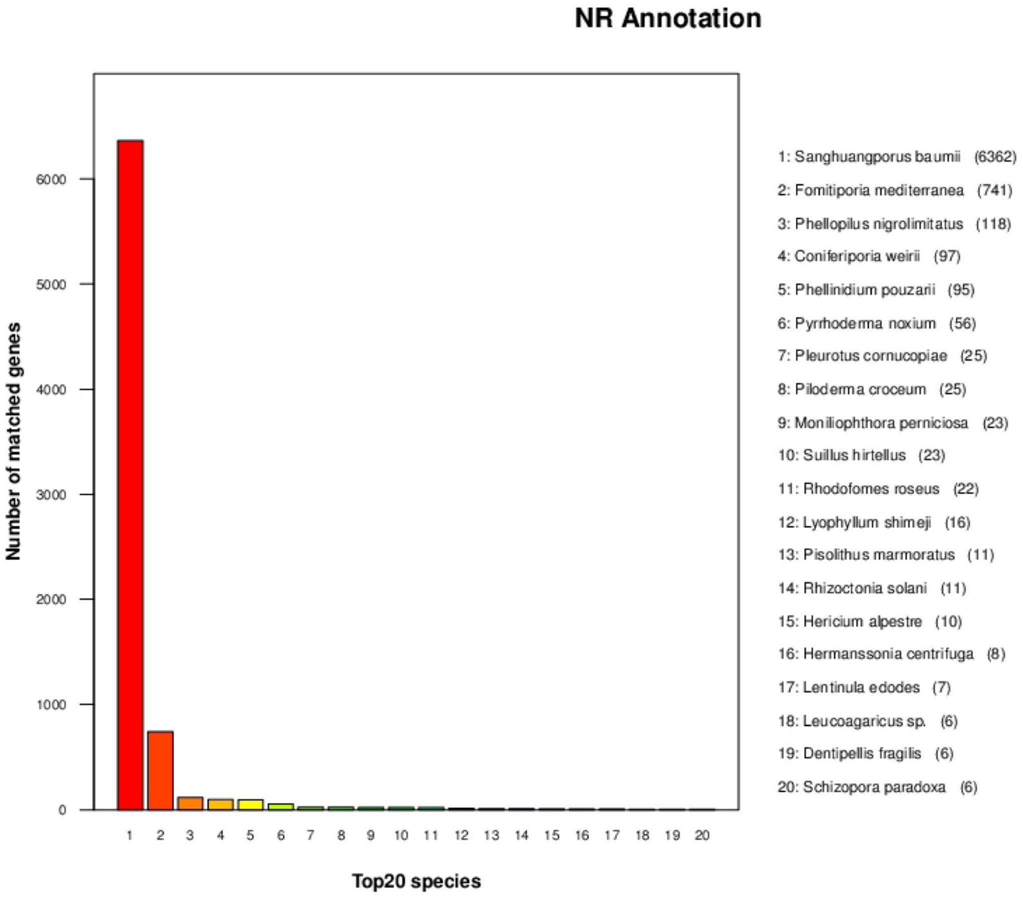

Figure S2. NR Annotation of *S. mongolicus*.

Table S1. Lengths of individual contigs in the final *S. mongolicus* genome assembly.

Contig	Length (bp)	Length (Mb)
Contig1	4,638,348	4.638
Contig2	4,205,964	4.206
Contig3	3,963,613	3.964
Contig4	3,180,165	3.180
Contig5	3,090,157	3.090
Contig6	2,836,961	2.837
Contig7	2,637,787	2.638
Contig8	2,360,954	2.361
Contig9	2,074,490	2.074
Contig10	1,732,583	1.733
Contig11	1,659,034	1.659
Contig12	1,609,254	1.609
Contig13	802,757	0.803

Table S2. Manual NCBI BLASTp verification of flavonoid- and phenylpropanoid-related candidate proteins identified in *S. mongolicus*.

Gene ID	Candidate annotation	Representative functionally annotated ClusteredNR hit	Organism	Accession	Query cover (%)	Identity (%)	E-value	Max score
A1670	Phenylalanine ammonia-lyase (PAL) candidate	Phenylalanine ammonia-lyase	*S. baumii*	OCB91043.1	100	96.16	0.0	1640
A5498	4-coumaroyl-CoA ligase-like (4CL-like) candidate	4-coumaroyl-CoA ligase	*S. baumii*	WBR81425.1	100	93.06	0.0	1107
A4080	Pirin-domain-containing protein candidate	Pirin-domain-containing protein	*S. baumii*	OCB86410.1	100	91.74	3×10^{-162}	467
A2183	GH78-related glycoside hydrolase candidate	Six-hairpin glycosidase	*S. baumii*	OCB86064.1	100	95.25	0.0	1309
A6830	GH78-related glycoside hydrolase candidate	Glycoside hydrolase family 78 protein	*S. baumii*	OCB87331.1	98	88.75	0.0	957

Note: Candidate proteins were manually examined using the NCBI Protein BLAST (BLASTp) web interface against the ClusteredNR protein database. Representative functionally annotated hits were selected from the highest-scoring matches. For candidates whose highest-scoring representative was annotated as a hypothetical protein, a highly ranked hit with an explicit functional annotation is reported. An E-value of 0.0 indicates an extremely small value displayed as zero by NCBI BLAST rather than a missing value. BLAST similarity supports candidate family-level annotation but does not constitute experimental confirmation of enzyme activity or substrate specificity.

Table S3. Comparison of genome features, repeat-sequence content, and selected functional annotations among five *Sanghuangporus* species.

Category	Feature	*S. baumii*	*S. sanghuang*	*S. vaninii*	*S. weigelae*	*S. mongolicus*
Genome features	Genome size (Mb)	31.64	33.34	34.52	33.96	34.79
Genome features	Number of contigs	339	26	37	13	13
Genome features	Contig N50 (Mb)	0.18	2.06	2.02	2.77	3.09
Genome features	Protein-coding genes	8455	8278	11310	9377	8471
Genome features	Gene density (genes/Mb)[a]	267.2	248.3	327.6	276.1	243.5
Genome features	Reported mean gene length (bp)[b]	NR	1,597	1,930	2,061	1,611
Genome features	GC content (%)	47.25	48.04	47.95	47.93	48.18
Repeat-sequence content [c]	LTR retrotransposons (%)	NR	2.72	9.84	12.22	2.9610
Repeat-sequence content [c]	DNA transposons (%)	NR	NR	NR	1.76	0.0671
Repeat-sequence content [c]	LINEs (%)	NR	NR	NR	0.30	0.3099
Repeat-sequence content [c]	SINEs (%)	NR	NR	NR	0.01	0.0041
Repeat-sequence content [c]	Rolling-circle repeats (%)	NR	NR	NR	0.00	0.0039
Repeat-sequence content [c]	Unclassified repeats (%)	NR	NR	NR	1.98	0.0022
Repeat-sequence content [c]	Dispersed repeats (%)	NR	2.90	NR	NR	3.3262
Repeat-sequence content [c]	Tandem repeats (%)	NR	0.85	0.27	0.27	0.8270
CAZyme-related annotations [d]	CBM	39	38	38	38	56
CAZyme-related annotations [d]	CE	15	23	18	23	26
CAZyme-related annotations [d]	GH	155	162	160	169	210
CAZyme-related	GT	54	62	61	50	123

annotations [d]						
CAZyme-related annotations [d]	PL	5	9	8	6	12
CAZyme-related annotations [d]	AA	45	52	53	46	64
CAZyme-related annotations [d]	Total	313	346	338	332	491
Normalized CAZyme metrics [e]	CAZyme annotations per Mb[c]	9.89	10.38	9.79	9.78	14.11
Normalized CAZyme metrics [e]	CAZyme annotations per 100 predicted protein-coding genes[d]	3.70	4.18	2.99	3.54	5.80
Predicted CYP genes	B-class P450	1	0	0	0	0
Predicted CYP genes	Cytochrome P450	8	12	9	8	7
Predicted CYP genes	E-class P450, CYP2D	1	1	1	1	1
Predicted CYP genes	E-class P450, group I	68	66	80	65	71
Predicted CYP genes	E-class P450, group IV	7	8	14	20	9
Predicted CYP genes	P450, CYP52	5	5	6	5	4
Predicted CYP genes	Pisatin demethylase-like	4	6	2	8	3
Predicted CYP genes	Undetermined	18	25	24	23	28
Predicted CYP genes	Total	112	121	136	130	123
Predicted secondary metabolite biosynthetic gene clusters	Terpene	12	10	11	9	11
Predicted secondary metabolite biosynthetic gene	T1PKS	1	4	1	3	1

clusters						
Predicted secondary metabolite biosynthetic gene clusters	NRPS	3	1	3	4	1
Predicted secondary metabolite biosynthetic gene clusters	NRPS-like/T1PKS	0	0	0	0	2
Predicted secondary metabolite biosynthetic gene clusters	NRPS-like	0	0	0	0	1
Predicted secondary metabolite biosynthetic gene clusters	Other molecules	2	1	1	3	0
Predicted secondary metabolite biosynthetic gene clusters	Total	18	16	16	19	16

Notes: Values for the four previously published genomes were obtained from the corresponding studies, unless otherwise stated. Functional annotations represent computational predictions.

Abbreviations: AA, auxiliary activities; CAZyme, carbohydrate-active enzyme; CBM, carbohydrate-binding module; CE, carbohydrate esterase; CYP, cytochrome P450; GH, glycoside hydrolase; GT, glycosyltransferase; LINE, long interspersed nuclear element; LTR, long terminal repeat; MITE, miniature inverted-repeat transposable element; NR, not reported; NRPS, nonribosomal peptide synthetase; PL, polysaccharide lyase; SINE, short interspersed nuclear element; T1PKS, type I polyketide synthase.

[a] Gene density was calculated as the number of predicted protein-coding genes divided by genome size in megabases.

[b] Mean gene length is shown using the terminology and values reported in the original studies. Because annotation pipelines differed, these values are intended for descriptive comparison and do not directly validate individual gene models. A consistently defined gene-length-to-genome proportion was unavailable for the published congeners and was therefore not included.

[c] Repeat-sequence proportions were obtained from the corresponding original studies and are shown at the

precision available in each source. Differences in annotation pipelines, databases, and classification criteria limit direct comparison. These values are therefore presented descriptively. The *S. weigelae* DNA-transposon value includes DNA/TcMar, DNA/TcMar-Ant1, DNA/others, and MITE categories. For *S. mongolicus*, dispersed and tandem repeats were detected using different procedures and are reported separately. A nonredundant total repeat proportion was therefore not calculated.

[d] CAZyme-related annotation counts for *S. baumii*, *S. sanghuang*, *S. vaninii*, and *S. weigelae* were obtained from Jin et al. The first three genomes were reannotated using the pipeline applied to S. weigelae. For S. mongolicus, predicted proteins were searched against CAZy using DIAMOND. Hits required an E-value $\leq 1 \times 10^{-5}$, sequence identity ≥ 40%, and alignment coverage ≥ 40%. Only the best qualifying hit was retained for each query. However, structural gene-prediction workflows differed between the present study and Jin et al. Accordingly, the counts are presented descriptively and do not constitute formal evidence of gene-family expansion.

[e] CAZyme annotations per megabase were calculated by dividing the total number of CAZyme-related annotations by genome size. Values per 100 predicted protein-coding genes were calculated by dividing the total annotation count by predicted gene number and multiplying by 100. Each species was represented by one genome, and annotation counts were deterministic outputs rather than biological replicates. Consequently, normalized values were compared descriptively, without inferential significance testing.
